# Surgical Management of Gallstone Ileus in Low-Settings Hospital during COVID-19 Outbreak: A Case Report

**DOI:** 10.1055/s-0041-1725160

**Published:** 2021-05-25

**Authors:** Ghaith Al-Abbasi, Ali Adil Alhilfy, Ameer Al-Jasim

**Affiliations:** 1 University of Baghdad, Baghdad, Iraq

**Keywords:** surgical management, gallstone ileus, COVID-19, low-settings hospital, case report

## Abstract

**Introduction** Gallstone ileus is a very infrequent complication of cholelithiasis in which single or multiple stones pass through an abnormal fistula to the lumen of the intestine leading to a true mechanical obstruction. We are reporting a case of a female who developed intestinal obstruction due to gallstones during the coronavirus disease 2019 (COVID-19) outbreak and was managed urgently surgically in a low-settings hospital.

**Case Presentation** An 85-year-old white female with 40 years history of gallstone disease, hypertension, and type-2 diabetes presented to the accidents and emergency unit with upper central crampy abdominal pain for 5 days associated with green color vomiting and absolute constipation. On examination, she was barely stable, dehydrated, had a distended abdomen, and guarding in the epigastric region. Her electrolytes were disturbed and had elevated serum creatinine and blood urea. Imaging studies confirmed gallstone ileus. Management was surgical despite the lack of facilities and equipment including COVID-19 personal protective equipment.

**Conclusion** Despite being an infrequent complication, gallstone ileus might present at the most unexpected time and in the least equipped hospital where the surgeon's suspicion, risk stratification, and improvisation by utilizing what is available are the keys for successful management and saving lives.

Gallstone ileus is the condition in which single or multiple stones of different sizes pass through a cholecysto-intestinal fistula and impacted in the lumen of the intestine leading to mechanical obstruction. It is a very infrequent complication of cholelithiasis where only 0.3 to 0.5% of patients with cholelithiasis will develop gallstone ileus. The term “ileus” is a misnomer and has no role in the pathophysiology as the obstruction is a true mechanical one. 1

Gallstone ileus poses a diagnostic and therapeutic challenge to the general surgeons practicing in low-settings hospitals since major bowel surgery in the settings of emergency has one of the highest mortalities, which can reach 50% in the over 80s; many of the patients undergoing this type of surgery are elderly, have multiple comorbidities, and need special postoperative care including intensive care unit (ICU), high dependency unit (HDU), and post anesthesia care unit (PACU). 2 Furthermore, rural hospitals in Iraq usually lack the robust infection prevention and control programs along with the absence of nationwide surveillance systems. As we are facing the global pandemic of coronavirus disease 2019 (COVID-19), Centers for Disease Control (CDC) guidelines for environmental infection control in health care facilities are not met due to the lack of resources. For example, telemedicine services are not implemented and health services were provided based on of first come first served, thus overcrowding in the clinics and wards is not uncommon with limited hospital beds leading to sharing of beds and placing patients on the floors in the surrounding hallways and stairways. Screening stations around the hospital were composed of a portable infrared forehead thermometer with a sensitivity of 63.7%. In terms of hospital structure, continuous negative-air-pressure, well-sealed rooms, and self-closing devices are not available to reduce the hospital-acquired infections as recommended by the CDC. 3

The standard management of gallstone ileus also remains disputed whether enterotomy alone (one-stage procedure) or in combination with cholecystectomy and fistula closure. The dilemma was which choice to go with putting in mind the resource-limited environment.

In this case, we report a female patient who developed intestinal obstruction due to gallstones during COVID-19 outbreak and was urgently managed surgically in a low-settings hospital.

## Case Description

An 85-year-old white female with a history of gallstone disease (40 years ago), hypertension, and type-2 diabetes presented to the accidents and emergency unit (A&E) with upper central crampy abdominal pain for 5 days. The pain was severe enough to interfere with her daily life. It was aggravated by eating with mild relief felt after vomiting. The vomitus was green in color and continuous throughout the whole day. The patient reported absolute constipation as well. The patient did not have any history of surgeries.

On physical examination, she was conscious, alert, lying supine in bed, seemed in pain, and was dehydrated. Her body mass index was 28.8. There was no jaundice or pallor. Vital signs were temperature 37°C, pulse 110, respiratory rate 14, blood pressure 140/80. The abdomen was distended symmetrically and moved freely with respiration. No scars were noted. Palpation revealed guarding in the epigastric region. No masses or organomegaly were identified. The abdomen was tympanic on percussion. Shifting dullness and transmission thrill were all negative. Auscultation revealed high pitched tinkling bowel sounds with a rate of 10 per minute. Digital rectal examination revealed empty rectum. The hernial sites were free and there was no lymphadenopathy.

Laboratory results showed disturbed serum electrolytes (Na ^+^ , K ^+^ , Cl ^−^ ), serum creatinine 2.7 mg/dL, blood urea 280 mg/dL, total serum bilirubin 0.8 mg/dL, hemoglobin 13.7 g/dL, white blood cell count 110 K/cmm, and platelet count of 250 K/cmm.

**Fig. 1:**
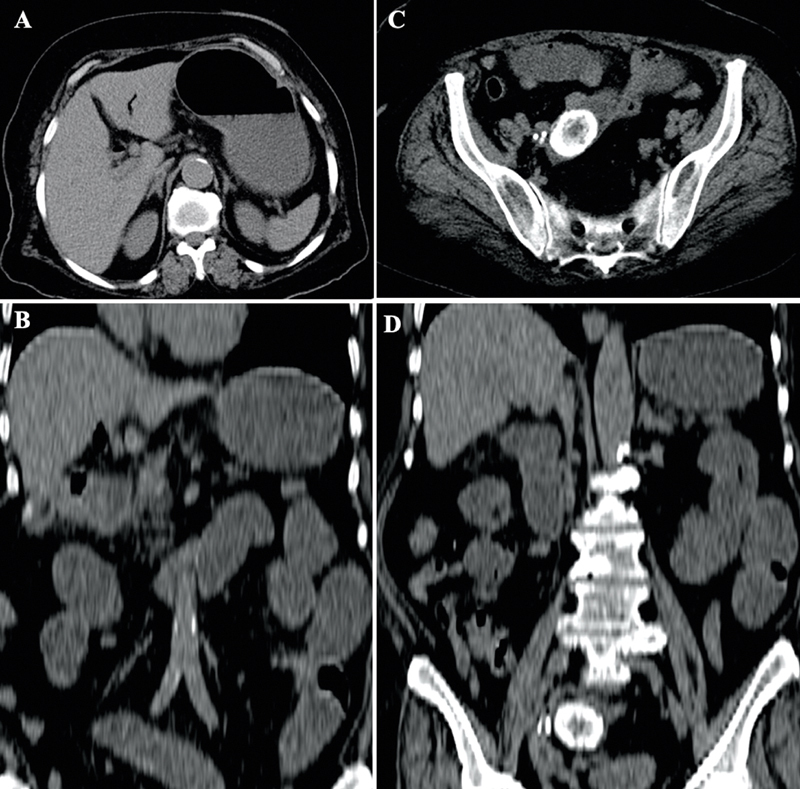
Preoperative CT scan of the abdomen showing pneumobilia ( **A** ), cholecystoenteric fistula between gallbladder and duodenum ( **B** ), and multiple stones in proximal ileum with bowel dilation ( **C, D** ).

A plain abdominal radiograph revealed dilated bowel loops with multiple air-fluid levels. Ultrasonography was done bedside and showed multiple stones in the gallbladder, air in the biliary tree (pneumobilia), and adhesion of the duodenum to the gallbladder. Besides that, abdominal CT scan was done and showed pneumobilia ( Fig. 1A ), cholecystoenteric fistula (gallbladder and duodenum) ( Fig. 1B ), and different size stones in which the largest one measuring 3.5 cm in the proximal ileum with bowel dilation proximal to them ( Fig. 1C and D ).

Ideally, patients should be further tested for COVID-19 before the surgery. However, there were no testing kits available at the hospital for such a purpose.

The nasogastric tube which was inserted in the A&E drained 1,500 mL of bilious fluid. Then the patient was admitted to the surgical ward for observation and correction of dehydration and renal impairment to be prepared for the surgery.

**Fig. 2:**
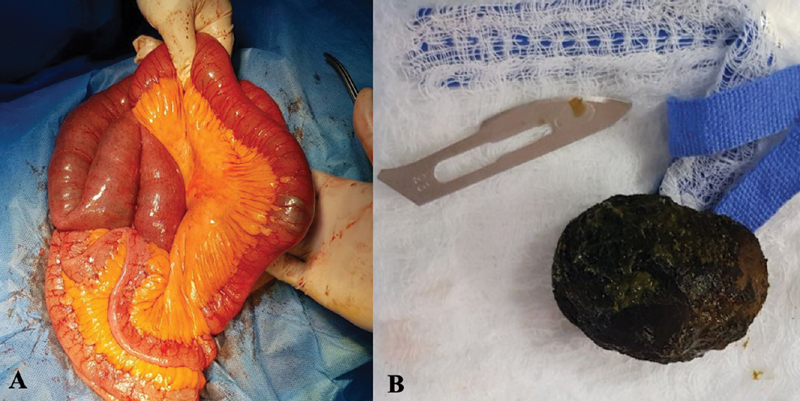
The stone before ( **A** ) and after ( **B** ) extraction.

The patient was then taken up for urgent surgery within less than 48 hours from admission. This is after managing her reversible acute prerenal impairment with good hydration. Then, she was taken to the operation room when her renal indices (blood urea and serum creatinine) returned to the normal range. COVID-19 precautions including personal protective equipment were not available for the surgical team at the time of the operation. Therefore, surgery was performed with the least level of precautions available. Since laparoscopy was not available at our hospital, open surgery was the choice. During exploration, scanty amount of free serosal fluid was noticed in the peritoneal cavity along with dilated small bowel loops. Three centimeters and a half-sized gallstone was found obstructing the proximal ileum 200 cm before Bauhin's valve ( Fig. 2A ). Consequently, longitudinal enterotomy was done and the stones were all extracted and the bowel was then closed transversally in two layers. The surgery was accomplished with no recorded complications in 60 minutes. Due to the patient's general condition, lack of ICU services at the hospital, severe inflammation, and associated adhesions surrounding the gallbladder, cholecystectomy and fistula closure was deferred to a later date. The follow-up and recovery were uneventful. She was discharged home well on postoperative day four. She was scheduled for multiple follow-up visits to decide a further management plan considering a referral to a tertiary center (after which COVID-19 situation is better controlled in the country) where both needed facilities and expertise are available.

## Discussion

Intestinal obstruction has always been a diagnostic and therapeutic dilemma in low-settings hospitals. In rural Africa, acute intestinal obstruction accounts for a great proportion of morbidity and mortality. For example, in Ethiopia, the prevalence of intestinal obstruction is 4.8% among surgical cases, and 21.8% among patients admitted with acute abdomen. 4 Intravenous fluids and electrolytes, gastrointestinal aspiration, antibiotics, and timed appropriate surgery are still the mainstay of treatment in rural areas. The commonest specific type of intraoperative procedure done, after a general laparotomy, to treat the patients with intestinal obstruction was resection and anastomosis. 5 The outcome measures vary according to the patients' factors and hospital settings. One of the important factors determining the surgical outcome is the duration of illness before surgical intervention. Patients who presented within 24 hours duration of illness are less likely to develop unfavorable outcome compared with patients who presented after 24 hours. 4 Hospital settings that play a role in the outcomes of surgical intervention include the availability of ICU, HDU, and PACU especially for elderly patients with multiple comorbidities in whom the operation poses a major risk on their lives. These settings, unfortunately, are rarely provided in rural hospitals despite being in need leading to an impact on the overall surgical outcome.

Gallstone is an infrequent cause of intestinal obstruction. Still, it stands for 25% of nonstrangulated bowel obstruction cases in elderly who need special perioperative care including ICUs. 6 7

Usually, acute cholecystitis precedes and adhesion supervenes. Fistula formation develops later when the gallstone stresses enough to cause erosion through the gallbladder wall to open a tract that connects the gallbladder with the stomach, small bowel or transverse colon into which the stone passes. The duodenum is the most commonly reported site of fistula due to its anatomical proximity. 1

Apart from a clinical diagnosis of intestinal obstruction, gallstone ileus can be confirmed by further radiologic evaluation, more specifically “Rigler's triad” on CT scan, a combination of pneumobilia, small bowel obstruction, and ectopic stone(s). However, a clear and straight forward triad is uncommon and found only in 9 to 14% of cases (our case belongs to them) rendering the diagnosis undoubtful. 8

The management of gallstone ileus worldwide is still being controversial because some surgeons performed several procedures like laparoscopy, lithotripsy, and others as an alternative to open surgery. 9 For open surgery many options are available including simple enterolithotomy, cholecystectomy and fistula closure (one-stage procedure), and enterolithotomy with cholecystectomy performed later (two-stage procedure). Nevertheless, simple enterolithotomy is the most commonly reported surgery done. 1

Because gallstone ileus is more common in elderly (who need special perioperative care including intensive care services) 7 and no significant changes in the mortality rate and length of stay of gallstone ileus patients over the past 25 years were documented despite the development in medicine, 10 it was never published previously that such infrequent complication was managed in a low-settings hospital during COVID-19 outbreak.

In this case, admitting the patient to the hospital, in the first place, was difficult as the hospital had sustained an unfortunate fire accident rendering it functional with only one ward shared between medical and surgical departments with only 20 beds assigned for surgical patients out of which most of them were occupied with patients by that time.

The decision of whether or not to operate at our hospital was challenging as well. The worst-case scenario that we had in mind was that the patient might need admission to respiratory care unit postoperatively which—along with other facilities—was missing in our hospital. Instead, there were four functioning ventilators available for the surgical and nonsurgical patients (apart from operation room ventilators). They were all occupied with very critical patients by that time. Therefore, the plan was to keep the patient intubated in the operation room in case she needed to.

Vitals were barely stable after initial management. Transferring her to another province for a better hospital with good respiratory, intensive care units, and COVID-19 precautions was not applicable due to lockdown and internal borders closure by the government in response to COVID-19 outbreak. Putting in mind that Iraq lacks the facilities of air medical services, management had to be done promptly at our low-settings hospital after weighing the risks of both clinical deterioration and the possibility of contracting COVID-19 infection with the benefits of clinical improvement, furthermore, patient was informed about the risks of the operation and her perspective was to follow through with what the surgeon advises as long as he is confident of that.

Upon planning for the operation, we considered the availability of the facilities and calculated the risk against the benefits for choosing the most appropriate plan, and since the duration of abdominal surgery is significantly associated with postoperative pulmonary and cardiac complications, we decided that simple enterolithotomy would be the life-saving procedure for the patient at the meantime. 11

This case report adds to the evidence that gallstone ileus although being infrequent, surgeons must have high suspicion to diagnose and deal with such complication to save patients' lives as it might present at the most unexpected time in the least equipped hospital. Therefore, we suggest further studies focusing mainly on risk stratification and assessment for patients present with gallstone ileus in which the choice of procedure and the decision to refer the patient—if possible—would be easier and universal.
